# Incidence of *Echinococcus granulosus* in Domestic Dogs in Palestine as Revealed by Copro-PCR

**DOI:** 10.1371/journal.pntd.0003934

**Published:** 2015-07-16

**Authors:** Amer Al-Jawabreh, Kamal Dumaidi, Suheir Ereqat, Abedelmajeed Nasereddin, Hanan Al-Jawabreh, Kifaya Azmi, Nahed Al-Laham, Ziad Abdeen

**Affiliations:** 1 Arab American University in Jenin, Jenin, Palestine; 2 Al-Quds Public Health Society, Jerusalem, Palestine; 3 Department of Biochemistry and Molecular Biology, Faculty of Medicine, Al-Quds University, East Jerusalem, Abu-Deis, Palestine; 4 Al-Quds Nutrition and Health Research Institute, Al-Quds University, East Jerusalem, Palestine; 5 Department of Laboratory Medicine, Faculty of Applied Medical Sciences, Al Azhar University–Gaza, Gaza Strip, Palestine; The First Affiliated Hospital of Xinjiang Medical University, CHINA

## Abstract

Hydatidosis or echinococcosisis considered a neglected zoonotic disease despite its high burden in the livestock industry and the high risk of infection by humans in endemic areas. In a cross-sectional study we estimated the copro-Incidence and also genotyped *Echinococcus granulosus* isolates from domestic dogs using polymerase chain reaction (PCR). Medical archives in nine major hospitals in Palestine were reviewed to determine incidence of *E*. *granulosus* infection detected in humans during surgery. Faecal samples were collected from 93 domestic dogs in three districts with the highest number of human cases: Al-Khalil (Hebron), Tubas and Jenin. Genomic DNA was extracted from dog faecal samples and amplified by PCR targeting the repeat DNA sequence (EgG1 Hae III) followed by sequencing of five positive samples. Genotyping was determined by sequencing and BLAST searching of mitochondrial cytochrome c oxidase subunit (CO1). The incidence of *E*. *granulosus* infection detected in humans at surgery was 1.2 per 100,000 in the West Bank and 1.0 per 100,000 in Gaza Strip. Seventeen of 93 domestic dogs (18%) were positive, based upon comparison with the *Echinococcus* DNA control. The five sequenced samples were confirmed to be *E*. *granulosus*. Successfully genotyped sample belonged to *E*.*granulosus sensu stricto* (formerly G1-G3 complex, sheep strain). For domestic dogs, age group (13-24 months) and sex were identified as two risk factors for contracting *E*. *granulosus*. The study identified the high incidence of *E. granulosus sensu stricto* in dogs in Palestine.

## Author Summary

Echinococcosis is a parasitic disease caused by *Echinococcus granulosus*. The parasite life cycle is shared between a definitive and an intermediate host with humans as accidental hosts. The dog is a main definitive host while sheep is a major intermediate host. The incidence of *E*. *granulosus* infection detected in humans at surgery is a commonly used parameter to evaluate the incidence rate of the disease in Palestine. However, this parameter of *Echinococcus* detection leads to under-reporting of the infection. This study aims to evaluate the incidence of *E*. *granulosus* infection in domestic dogs as a potential source of infection to human using molecular methods. Medical archives in nine major hospitals in Palestine were reviewed for *E*. *granulosus* infection detected during surgery. Faecal samples were collected from 93 domestic dogs in areas with the highest levels of *E*. *granulosus* infection detected in humans at surgery in southern Palestine, including Al-Khalil, and northern Palestine, including Tubas and Jenin districts. The overall incidence rate of *E*. *granulosus* infection detected in humans during surgery was 0.9 per 100,000. Around 18% (17/93) of the dogs were infected. Results that *E*. *granulosus* was the causative agent were confirmed by sequencing and BLAST search of five positive samples. Genotyping of one sample showed that infection was caused by *E*. *granulosus sensu stricto*. The age and sex of dogs were identified as risk factors for contracting infection.

## Introduction

*Echinococcus granulosus*, causing hydatid disease (hydatidosis), is a taeniid cestode infecting dogs and other canidae as definitive hosts and herbivores, for example sheep, goats, horses, camels, cattle and pigsas intermediate hosts. The dog ingests the infected organs of the intermediate host such as liver and lungs which contain hydatid cyst with protoscolices. Humans are considered as an accidental intermediate host in which echinococcosis (alveolar, cystic or polycystic) develops following human ingestion of eggs of *Echinococcus* species shed from the feces of dogs harboring adult stages of the tapeworm. This zoonosis has been recognized by the WHO as one of the seventeen neglected diseases in the world, with more than one million human cases at any given time [1]. Hydatidosis, of which 95% is cystic echinococcosis (CE),is widely spread in all Mediterranean basin countries with incidence determined in humans at surgery ranging from 0.28 in France to 15 cases per 100,000 inhabitants in Tunisia [2,3]. In highly endemic countries as in China, central Asia and South America prevalence may reach as high as 50 per 100,000 persons with CE in slaughtered livestock ranging between 20 to 95% [4].

In Palestine, hydatidosis is a reportable disease appearing in the Ministry of Health annual report[5]. A study in 2002 revealed the sero-prevalence of CE among school children in Palestine to be 2.4% [6]. A study in the Nagab area in and around the city of Rahat showed that sero-prevalence among human subjects was 1.5 per 100,000 persons[7]. The genotyping of isolated *Echinococcus granulosus* strains is of paramount importance due to difference in life cycles, parasite transmission patterns, host susceptibility to different genotypes, different clinical picture in terms of the size of cyst and severity as well as geographical distribution of each genotype [8,9].

The genus *Echinococcus* contains 9 validated species with genotypes G1 to G10 [10]. *E*. *granulosus* has been characterized to have ten genotypes (G1–G10), based on mitochondrial sequences such as cytochrome c oxidase (CO1) with *E*. *granulosus senso stricto* (formerly sheep strain, G1–G3) forming 90% of the isolates [10,11]. Hydatidosis is mainly associated with risk factors such as the livestock production and breeding, dog care and behavior as well as life style and health awareness of human host [12].

The aim of this study was to determine the incidence of *E*. *granulosus* infection in domestic dogs using copro-PCR and the association with risk factors in Palestine. Furthermore, we aim to identify the most prevalent *E*. *granulosus* genotype infecting the dogs.

## Materials and Methods

### Study design

**Fig 1:**
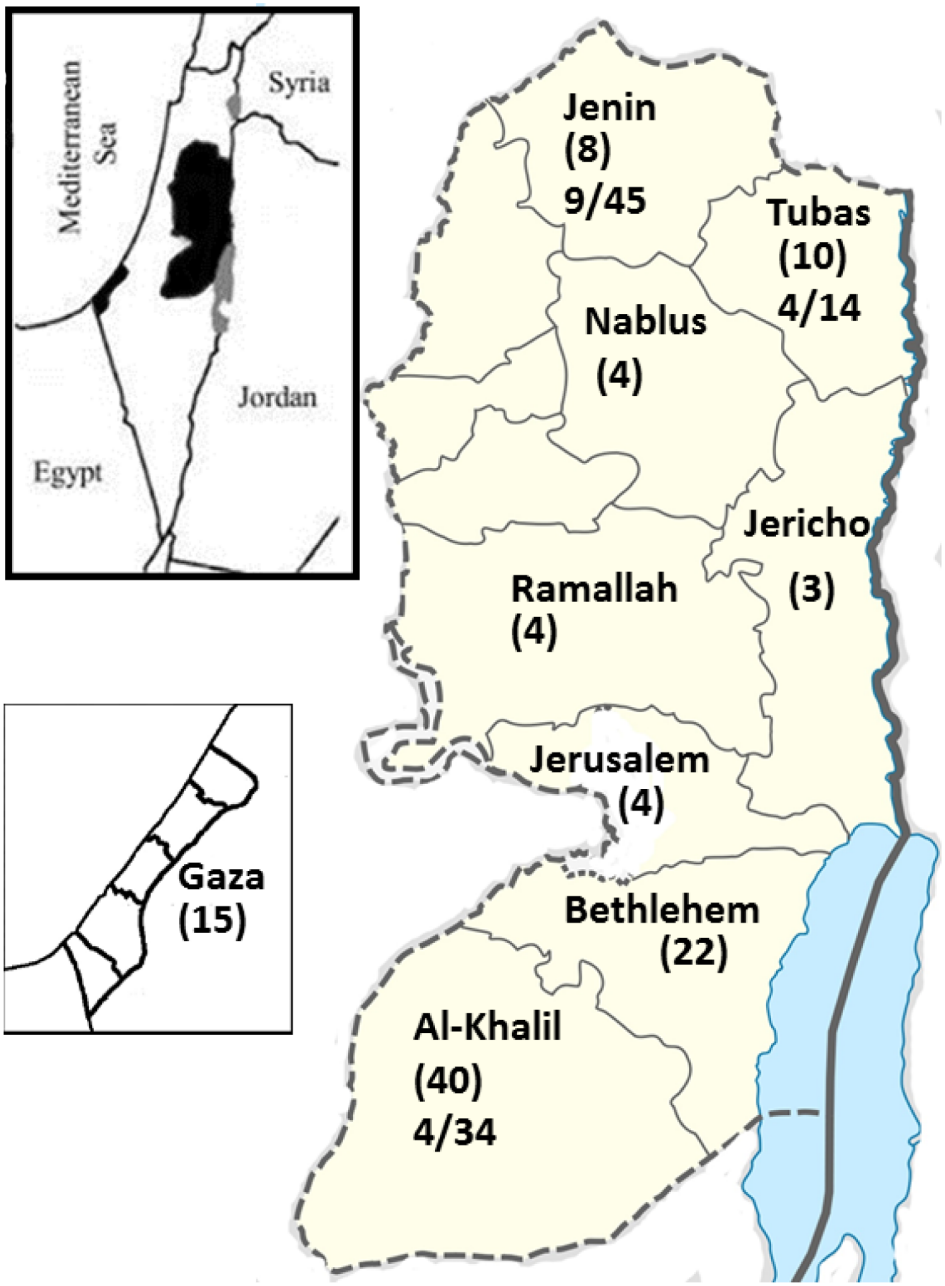
Distribution of sampled domestic dogs: Map of Palestine West Bank and Gaza showing in brackets the number of human cases reviewed in the medical archives and the three districts from which dog fecal samples were collected. First number indicates positive results while the second one represents the total number of sampled dogs.

A cross-sectional pilot study on *E*. *granulosus* infection in domestic dogs was conducted in the northern and southern Palestinian districts with the highest numbers of human echinococcosis (hydatid cyst disease) cases in the years 2013 and 2014 (Fig 1).

### Medical records review

Medical records of the CE patients in nine major hospitals were reviewed from the past 3 to 8 years depending on availability of data. The hydatid cyst case definition adopted and practiced in the hospitals depended mainly on radiography in the pre-operative stage and confirmed post-operatively by histopathology. The hospitals included Jenin Government Hospital, Rafidia Government Hospital in Nablus, Al-Khalil Government Hospital, Al-Ahli Hospital in Al-Khalil (Hebron), Al-Mezan Hospital in Al-Khalil (Hebron), Beit-Jala Government Hospital in Bethlehem, Jericho Government Hospital, Al-Makassed Hospital in East Jerusalem (Al-Quds) and Gaza European Hospital in Rafah (Fig 1). These hospitals are the main reference hospitals for surgery in Palestine. Data were analyzed to estimate the incidence of *E*. *granulosus* infection detected in humans during surgery in Palestine.

### Collection of faecal samples

In 2014, faecal samples were collected from dogs in two cities and 7 villages in northern and southern districts with highest numbers of human CE cases (Fig 1). Dogs around houses were caught in the evening, tied and fresh faecal samples collected next day in a labeled plastic container. Samples were transported to the laboratory in Jericho and kept at -70°C until used. A questionnaire on the dogs’ general health and behavior status was filled by directly interviewing the owner. The questionnaire included data about demography of the dog, for example, age, sex, color and breed. Dog behavior was also recorded, including types and sources of food, being loose, time spent with stray dogs, proximity of shelter from house, contact with children, and association with livestock. Veterinary care and any apparent disease symptoms were also recorded. Dog sampling was undertaken in areas where the human incidence of hydatid cyst infection was considered to be high in the northern and southern parts of Palestine.

### Molecular assays

#### Genomic DNA extraction

The echinococcal gDNA was extracted from faecal samples manually. Briefly, frozen faecal sample was thawed at room temperature and 0.5–1 g put into a 2- ml Eppendorf tube. Equal volumes of distilled water were added and vortex mixed for 1 min to make a homogenous faecal suspension. Prior to vortex mixing, 0.5 mm glass beads were added to increase efficiency of extraction by crushing. About 200 μl of the faecal suspension was transferred to a new 2-ml Eppendorf tube. A volume of 0.5 ml of TESP lysis buffer (100 mMTris-HCl [pH 8], 50 mM EDTA [pH 8], 10% SDS, 1% polyvinylpyrroli edone PVP) was added. Around 20 μl (2%) of 2-mercaptoethanol were added and incubated at 65°C for 90 min. Then, 20 μl of proteinase K (20 mg/ml; Sigma-Aldrich, St. Louis, MO) were added and incubated at 65°C for 2 hrs to overnight. Phenol-chloroform-isoamyl treatment and ethanol precipitation were carried out. The DNA pellet was then purified using commercially available kit Nucleospin Extract 2 in 1 from Macherey-Nagel-Germany (www.mn-net.com). DNA concentration and quality, A_260_/A_280_ ratio were measured by spectrophotometer (Nanodrop 2000C, Thermo Fisher Scientific-USA).

### Copro-PCR and sequence analysis

The echinococcal gDNA was amplified using PCR with two primer pairs, the 133 bp for diagnosis using primers Eg1121a and Eg1122a and the 269bp for sequence purposes using primers Eg2691 and Eg2692 targeting repeated DNA sequence (EgG1 Hae III), as described by others with slight modification[13]. Briefly, three μl of gDNA template were amplified in a 25 μl using PCR-ready Supreme mix (Syntezza Bioscience-Jerusalem). DMSO (0.625 μl, 2.5%) as PCR enhancer was added. One μl of each primer reaching a final concentration of 0.4 μM were added to the mix. Thermocycling profile was run as described by Abbasi *et al*. using Biorad C1000 thermocycler [13]. Positive (DNA extracted from human cyst) and negative controls (nuclease-free water) were used in each run. The PCR products were visualized by electrophoresis on 1.4% agarose gel in TBE buffer stained with ethidium bromide. For confirmation, all positive samples for 269 bp PCR system were sequenced for fragment of repeated *Hae III* region using primers (Eg2691 and Eg2692). Sequences were BLAST searched against the NCBI (National Center for Biotechnology Information, U.S. National Library of Medicine, 8600 Rockville Pike, Bethesda MD, 20894 USA) database.

### Genotyping of *Echinococcus granulosus*

PCR-positive samples were subjected to PCR amplification of the mitochondrial cytochrome c oxidase 1 (CO1) gene using primers and conditions described elsewhere using 0.4 μM forward and reverse primers, JB3 and JB4.5[14]. Briefly, 3 μl DNA template were added to a 25-μl-reaction HotstarTaq master mixture using HotStarTaq polymerase and accompanying reagents (QIAGEN GmbH, Hilden, Germany). The thermal cycling conditions were as follows: 95°C for 15 min; 38 cycles at 95°C for 60 s, 48°C for 60 s, and 72°C for 60 s; and a final extension at 72°C for 5 min. A negative control (nuclease-free water) was included in each PCR reaction to detect contamination. CO1 amplicon was sequenced and BLAST searched against E. *granulosus* strains from the gene bank (www.ncbi.nlm.nih.gov).

### Ethics statement

The study was approved by the ethics committee at the Faculty of Medicine in Al-Quds University-Palestine. All patients’ data in the archives of hospitals were kept private and anonymous using coding system for patients’ names. Dogs were not exposed to any invasive procedures. Samples were collected following normal defecation.

### Statistical analysis

Data were analyzed using Epi Info statistical package (CDC free-software). Contingency tables for infection outcome and potential exposure risk factors were tested using two-tailed Fisher’s exact test and chi square. The level of statistical significance was considered at P-value ≤ 0.05.

## Results

### Medical file review

A total of 110 patient files were reviewed in the surgical wards of the nine targeted hospitals in the last 3 years in the West Bank to 8 years in Gaza Strip. The cases were from 13 districts, 8 in the West Bank and 5 in Gaza Strip. About 25% (28/110) of the cases were from urban areas in 6 cities including Gaza Strip, while 75% were from rural areas in 27 villages in the West Bank (Table 1). Based on this file review, the faecal samples were collected from domestic dogs in Al-Khalil in the south, and Tubas and Jenin in the north (Fig 1).

**Table 1:** The incidence rate and number of *E*. *granulosus* infection cases detected in humans during surgery revealed by the study as compared to Ministry of Health official figures.

	Study		Ministry of Health
Area	Number of cases	3-year-mean incidence rate per 100,000§	5-year-mean incidence rate per 100,000*
The West Bank	95	1.2	0.8
Al-Khalil	40	1.9	3.4
Bethlehem	22	2.0	0.2
Tubas	10	5.6	1.8
Jenin	8	0.8	0.4
Nablus	4	0.3	2.4
Jericho (Ariha)	3	1.5	2.1
Al-Quds-Jerusalem	4	0.3	0.1
Qalqilia	0	0	0.1
Ramallah	4	1.3	0
Gaza	15	1 ‡	0.005
Palestine	110	0.9	0.8

^§^ 3-year-population average, 2011–2013.

*5-year-population average, 2009–2013.

^‡^8-year-population average, 2007–2014.

### Copro-PCR incidence rate and genotyping

**Fig 2:**
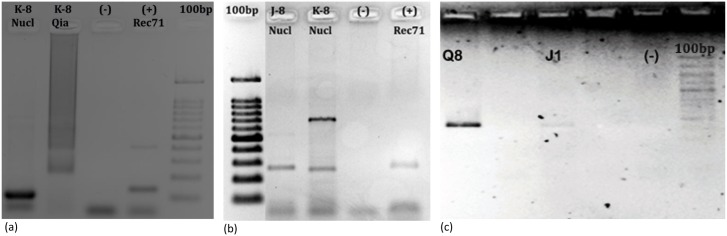
Results of copro-polymerase chain reaction with fecal samples from domestic dogs and controls. (a) Diagnostic 133 base pair-*Hae III* target (b) 269 base pair- *Hae III* target. J-8 and K-8 were shown to be *Echinococcus granulosus* by sequencing (c) 446 base pair- *CO1* target gene for genotyping. Sequencing of Q-8 PCR products revealed a G1 (sheep strain) genotype of *Echinococcus granulosus senso stricto*.

A total of 93 domestic dog faecal samples were collected from the three districts Al-Khalil, Jenin and Tubas. Of these, the faeces of 17 dogs were shown to have DNA of *E*. *granulosus* (18%) with a PCR product size of 133 bp as revealed by the *Hae III* repeat region amplification. Five of the 17 positive faecal samples were randomly selected from northern and southern Palestine for amplification and nucleotide-sequencing of the 269 bp product of the same region. BLAST search of the sequences in the GeneBank showed that all 5 were *E*. *granulosus*. The same 5 samples were amplified for the CO1gene (446 bp). The band with the highest density was subsequently nucleotide-sequenced. The BLAST search showed high similarity to *E*. *granulosus sensu stricto*, G1 sheep strain (GenBank Accession: KM100579.1) (Fig 2).

### Risk factors

All infected dogs were males, with faeces from no female dogs being positive (Table 2). Dogs in their second year of life are significantly more prone to infection than the rest of age groups. Although, over 70% (12/17) of infected dogs were from rural areas, there was no significant difference in *E*. *granulosus* infection rate between dogs living in urban areas and those from rural ones. The status of the dog being tied or loose did not significantly affect the infection rate. Around 55% (51/93) of the dogs accompanied the herds of livestock. Of the 17 infected dogs, 7 (41%) accompanied herds of sheep with no level of significance (P>0.05). The owners of 6(55%) of the 11 infected dogs, who were aware of the eating habits of their dogs indicated that their dogs ate remnants and offal of slaughtered livestock, particularly sheep. However, this was not statistically significant. Dogs that lived close to the house yard when not accompanying livestock were not significantly affect by *E*. *granulosus* infection. Out of 15 infected dogs, 11 (73%) played or lived in close proximity to children. Seven of the infected dogs were identified for their breed; 2 were German shepherds, 2 were from a local breed, while the rest were Huskys, and an Asian and a Caucasian shepherd.

**Table 2:** Demographic and behavioral factors associated with *E*. *granulosus* infection in domestic dogs in Palestine.

Variable		Positive	Negative	Total	Fisher’s exact test
Sex	Male	17	56	73	0.018*
	Female	0	20	20	
	Total	17	76	93	
Age group, months	0–12	5	31	36	0.028*
	13–24	9	16	25	
	>24	2	22	24	
	Total	16	69	85	
Tied vs loose	Tied	7	46	53	0.113
	Loose	2	2	4	
	Total	9	48	57	
Dog accompany livestock	Yes	7	44	51	0.16
	No	6	14	20	
	Total	13	58	71	
Source of food	Offal	6	36	42	0.75
	Other	5	24	29	
	Total	11	60	71	
Rural vs urban	Urban	5	10	15	0.13
	Rural	12	66	78	
	Total	17	76	93	

*Significant at P-value ≤ 0.05

## Discussion

This study was designed to provide an accurate estimate of hydatidosis in dogs in Palestine As surgery is the only treatment method for hydatid cyst disease in Palestine; medical files were reviewed in the nine major hospitals from patients treated in the past 3 years in the West Bank and in the past 8 years in Gaza Strip. This revealed that echinococcosis cases were mainly from Al-Khalil, Bethlehem, Jenin and Tubas. However, the disease is reported in all districts included in the study. Ministry of Health reported in the last five years (2009–2013) cases from 8 Palestinian districts with only one case from Gaza Strip[5]. The districts of Al-Khalil (Hebron), Nablus and Tubas were shown to have the highest 5-year-mean incidence of *E*. *granulosus* infection detected in humans at surgery, respectively[5]. The discrepancy between the Ministry of Health report and this study could be due to the case referral system which masked Jenin, Tubas and Bethlehem districts as echinococcosis hot spots. In light of the official figures and those from this study, the incidence of *E*. *granulosus* infection detected in humans at surgery in Palestine (0.9 per 100,000) is low compared to Jordan (2.3 per 100000), Egypt (2.6 per 100,000), and Turkey (6.4 per 100,000) [11,15,16].

The application of copro-PCR testing targeting repeated sequence of EgG1 *Hae III* region in the study detected a canine incidence of 18% in the three Palestinian districts, Al-Khalil, Jenin and Tubas. The relatively high incidence in dogs could be attributed to the limited access to veterinary care and improper feeding. However, the canine incidence rate in Palestine lies within the range reported by various studies in the region. Two Israeli studies among Palestinian residents in two towns in Galilee reported that 10.7% and 14.2% of the domestic dogs were infected [12,17]. In northern Jordan, one study put the infection rate among stray dogs as 13.8%, while the other showed 29.5% among semi-stray dogs [15,18]. In Egypt the infection rate among stray dogs was as low as 5% [19]. In Iran the rate among domestic dogs in east Iran was 23.7% and 18% in west Iran [20–22]. Of the five randomly-sequenced faecal dog samples for repeated *Hae III* region, one was sequenced for CO1 gene revealing the strains to be *E*. *granulosus sensu stricto*. Other studies in Palestine and the region including Jordan, Turkey and Iran were comparable, revealing *E*. *granulosus sensu stricto* as the predominant genotype in dogs and livestock [15,23,24].

Most of the study sample (78%) was male dogs, whilst all the 17 infected dogs were males. This indicates that dog keepers in Palestine prefer to raise male dogs rather than female, thus posing sex as a risk factor. Furthermore, the age of the dog is considered a significant risk factor. Dogs in their second year of life are more exposed to infection than older ones. A plausible explanation is that young male dogs are more active to wander thus increasing the probability of eating more infected remnants of slaughtered animal. A similar trend has been reported in neighboring countries such as Jordan and Egypt [15,19].

Although, hydatidosis or echinococcosis is a reportable disease in Palestine, little has been done to control the disease and there is insufficient awareness and attention from the official level as well as the general public. This is the first study in Palestine to tackle echinococcal infection in the definitive host, the domestic dog. The high *E*. *granulosus sensu stricto* infection rate in domestic dogs in Palestine is by default reflecting high infection rate in sheep and goats which is consequently forming a serious public health problem. A common practice among sheep and goat owners, which is considered a risk factor for spreading the disease, is feeding their dogs with the offal of their slaughter or disposing them on the outskirts of their villages for stray dogs to consume.

In the absence of data on rate of infection among livestock as intermediate host, variation between the incidence rate in humans (0.9 per 100,000) and the copro-PCR incidence rate among domestic dogs (18%) suggests under reporting and /or under diagnosis of human cases. This can be due to various reasons such as lack of public awareness, ineffective surveillance system and poor management information system (MIS). Archiving and MIS problems were readily noticed in the majority of the hospitals included in this study. This alarming rate of infection in domestic dogs requires diligent efforts by official bodies, represented by Ministry of Health and Ministry of Agriculture, and the non-official sectors such as non-governmental organization and private clinics. A strategy to control hydatid disease should be based on prompt medical intervention, screening programs, case-finding, and effective surveillance, active and passive case detection, as well as health awareness for dog keepers and livestock owners. The successfully-orchestrated strategy to control brucellosis in Palestine, which started in the year 2000 at an infection rate of 32 per 100,000 and ended up with a reduced rate of 3.4 per 100,000 in 2012 [5], provides an example for future action. This study gives a strong evidence of highly ongoing infection events in the definitive host which poses a great risk to humans.
